# Abdominal drainage after elective colorectal surgery: propensity score-matched retrospective analysis of an Italian cohort

**DOI:** 10.1093/bjsopen/zrad107

**Published:** 2024-01-03

**Authors:** Stefano Guadagni, Marco Catarci, Francesco Masedu, Mohammad Ehsanul Karim, Marco Clementi, Giacomo Ruffo, Massimo Giuseppe Viola, Felice Borghi, Gianandrea Baldazzi, Marco Scatizzi, Felice Pirozzi, Paolo Delrio, Gianluca Garulli, Pierluigi Marini, Roberto Campagnacci, Raffaele De Luca, Ferdinando Ficari, Giuseppe Sica, Stefano Scabini, Andrea Liverani, Marco Caricato, Alberto Patriti, the Italian ColoRectal Anastomotic Leakage (iCral) study group, Stefano Mancini, Gian Luca Baiocchi, Roberto Santoro, Walter Siquini, Gianluca Guercioni, Massimo Basti, Corrado Pedrazzani, Mauro Totis, Alessandro Carrara, Andrea Lucchi, Maurizio Pavanello, Andrea Muratore, Stefano D’Ugo, Alberto Di Leo, Giusto Pignata, Ugo Elmore, Gabriele Anania, Massimo Carlini, Francesco Corcione, Nereo Vettoretto, Graziano Longo, Mario Sorrentino, Antonio Giuliani, Giovanni Ferrari, Lucio Taglietti, Augusto Verzelli, Mariantonietta Di Cosmo, Davide Cavaliere, Marco Milone, Stefano Rausei, Giovanni Ciaccio, Giovanni Tebala, Giuseppe Brisinda, Stefano Berti, Paolo Millo, Luigi Boni, Mario Guerrieri, Roberto Persiani, Dario Parini, Antonino Spinelli, Michele Genna, Vincenzo Bottino, Andrea Coratti, Dario Scala, Umberto Rivolta, Micaela Piccoli, Carlo Talarico, Franco Roviello, Alessandro Anastasi, Giuseppe Maria Ettorre, Mauro Montuori, Pierpaolo Mariani, Nicolò de Manzini, Annibale Donini, Mariano Fortunato Armellino, Carlo Feo, Silvio Guerriero, Andrea Costanzi, Federico Marchesi, Moreno Cicetti, Paolo Ciano, Michele Benedetti, Leonardo Antonio Montemurro, Maria Sole Mattei, Elena Belloni, Daniela Apa, Matteo Di Carlo, Elisa Bertocchi, Gaia Masini, Amedeo Altamura, Francesco Rubichi, Desirée Cianflocca, Marco Migliore, Diletta Cassini, Lorenzo Pandolfini, Alessandro Falsetto, Antonio Sciuto, Ugo Pace, Andrea Fares Bucci, Francesco Monari, Grazia Maria Attinà, Angela Maurizi, Michele Simone, Francesco Giudici, Fabio Cianchi, Bruno Sensi, Alessandra Aprile, Domenico Soriero, Andrea Scarinci, Gabriella Teresa Capolupo, Valerio Sisti, Marcella Lodovica Ricci, Andrea Sagnotta, Sarah Molfino, Pietro Amodio, Alessandro Cardinali, Simone Cicconi, Irene Marziali, Diletta Frazzini, Cristian Conti, Nicolò Tamini, Marco Braga, Michele Motter, Giuseppe Tirone, Giacomo Martorelli, Alban Cacurri, Carlo Di Marco, Patrizia Marsanic, Nicoletta Sveva Pipitone Federico, Marcello Spampinato, Lorenzo Crepaz, Jacopo Andreuccetti, Ilaria Canfora, Giulia Maggi, Matteo Chiozza, Domenico Spoletini, Rosa Marcellinaro, Umberto Bracale, Roberto Peltrini, Maria Michela Di Nuzzo, Emanuele Botteri, Simone Santoni, Massimo Stefanoni, Giovanni Del Vecchio, Carmelo Magistro, Silvia Ruggiero, Arianna Birindelli, Andrea Budassi, Daniele Zigiotto, Leonardo Solaini, Giorgio Ercolani, Giovanni Domenico De Palma, Silvia Tenconi, Paolo Locurto, Antonio Di Cintio, Maria Michela Chiarello, Maria Cariati, Andrea Gennai, Manuela Grivon, Elisa Cassinotti, Monica Ortenzi, Alberto Biondi, Maurizio De Luca, Francesco Carrano, Francesca Fior, Antonio Ferronetti, Giuseppe Giuliani, Graziella Marino, Camillo Leonardo Bertoglio, Francesca Pecchini, Vincenzo Greco, Roberto Piagnerelli, Giuseppe Canonico, Marco Colasanti, Enrico Pinotti, Roberta Carminati, Edoardo Osenda, Luigina Graziosi, Ciro De Martino, Giovanna Ioia, Fioralba Pindozzi, Lorenzo Organetti, Michela Monteleone, Giorgio Dalmonte, Gabriele La Gioia

**Affiliations:** General Surgery Unit, University of L’Aquila, L'Aquila, Italy; General Surgery Unit, Sandro Pertini Hospital, ASL Roma 2, Roma, Italy; General Surgery Unit, ‘C.&G. Mazzoni’ Hospital, Ascoli Piceno, Italy; Department of Applied Clinical Sciences and Biotechnology, University of L’Aquila, L'Aquila, Italy; School of Population and Public Health, The University of British Columbia, Vancouver, BC, Canada; Centre for Health Evaluation and Outcome Sciences, St.Paul’s Hospital, Vancouver, BC, Canada; General Surgery Unit, University of L’Aquila, L'Aquila, Italy; General Surgery Unit, IRCCS Sacro Cuore Don Calabria Hospital, Negrar di Valpolicella (VR), Italy; General Surgery Unit, Cardinale G. Panico Hospital, Tricase, Italy; Oncologic Surgery Unit, Candiolo Cancer Institute, FPO-IRCCS, Candiolo, Italy; General & Oncologic Surgery Unit, Department of Surgery, Santa Croce e Carle Hospital, Cuneo, Italy; General Surgery Unit, ASST Ovest Milanese, Legnano, Italy; General Surgery Unit, ASST Nord Milano, Sesto San Giovanni, Italy; General Surgery Unit, Santa Maria Annunziata & Serristori Hospital, Firenze, Italy; General Surgery Unit, ASL Napoli 2 Nord, Pozzuoli, Italy; Colorectal Surgical Oncology, Istituto Nazionale per lo Studio e la Cura dei Tumori, ‘Fondazione Giovanni Pascale IRCCS-Italia’, Napoli, Italy; General Surgery Unit, Infermi Hospital, Rimini, Italy; General & Emergency Surgery Unit, San Camillo-Forlanini Hospital, Roma, Italy; General Surgery Unit, ‘C. Urbani’ Hospital, Jesi, Italy; Department of Surgical Oncology, IRCCS Istituto Tumori ‘Giovanni Paolo II’, Bari, Italy; General Surgery and IBD Unit, Careggi University Hospital, Firenze, Italy; Minimally Invasive Surgery Unit, Policlinico Tor Vergata University Hospital, Roma, Italy; General & Oncologic Surgery Unit, IRCCS ‘San Martino’ National Cancer Center, Genova, Italy; General Surgery Unit, Regina Apostolorum Hospital, Albano Laziale, Italy; Colorectal Surgery Unit, Policlinico Campus BioMedico, Roma, Italy; Department of Surgery, Marche Nord Hospital, Pesaro e Fano, Italy

## Abstract

**Background:**

In Italy, surgeons continue to drain the abdominal cavity in more than 50 per cent of patients after colorectal resection. The aim of this study was to evaluate the impact of abdominal drain placement on early adverse events in patients undergoing elective colorectal surgery.

**Methods:**

A database was retrospectively analysed through a 1:1 propensity score-matching model including 21 covariates. The primary endpoint was the postoperative duration of stay, and the secondary endpoints were surgical site infections, infectious morbidity rate defined as surgical site infections plus pulmonary infections plus urinary infections, anastomotic leakage, overall morbidity rate, major morbidity rate, reoperation and mortality rates. The results of multiple logistic regression analyses were presented as odds ratios (OR) and 95 per cent c.i.

**Results:**

A total of 6157 patients were analysed to produce two well-balanced groups of 1802 patients: group (A), no abdominal drain(s) and group (B), abdominal drain(s). Group A *versus* group B showed a significantly lower risk of postoperative duration of stay >6 days (OR 0.60; 95 per cent c.i. 0.51–0.70; *P* < 0.001). A mean postoperative duration of stay difference of 0.86 days was detected between groups. No difference was recorded between the two groups for all the other endpoints.

**Conclusion:**

This study confirms that placement of abdominal drain(s) after elective colorectal surgery is associated with a non-clinically significant longer (0.86 days) postoperative duration of stay but has no impact on any other secondary outcomes, confirming that abdominal drains should not be used routinely in colorectal surgery.

## Teaser

This large retrospective propensity score-matched analysis of a prospective cohort confirmed that there is no reason to support the still widespread use of abdominal drain(s) placement after elective colorectal resections.

## Introduction

More than 100 years after the statements of Robert Lawson Tait ‘When in doubt, drain’ and of William Stewart Halsted ‘No drainage at all is better than the ignorant employment of it’^1^, the assumption that the placement of peritoneal drains after elective colorectal surgery can provide diagnostic and therapeutic benefit through prevention and early detection of anastomotic leak or other intraperitoneal collections is debated^2,3^. Evidence suggests that drains can stimulate serous fluid production and may lead to an increased risk of surgical site infection (SSI)^4^ and adhesions, and prolonged hospital length of stay (LOS), impacting on postoperative pain control, mobility^4,5^, increased perceived discomfort and anxiety^6^. The Enhanced Recovery After Surgery (ERAS) Society^7^, the American Society of Colon and Rectal Surgeons and the Society of American Gastrointestinal and Endoscopic Surgeons^8^, French^9^ and Italian^10^ guidelines, based on RCTs^11,12^, older^13,14^ and more recent^15,16^ meta-analyses or systematic reviews of RCTs, strongly recommend that pelvic and peritoneal drains should not be used routinely in colorectal surgery. However, this strong recommendation is based on moderate-quality evidence^8,17^ (all the RCTs showed a bias of surgeon blinding, and some of them had a bias of allocation concealment and sequence randomization method^12^, systematic reviews/meta-analyses included a large number of infra-promontory anastomoses in which a pelvic drain is almost always placed) and mainly on data observed before the widespread application of minimally invasive surgery. Conversely, many surgeons, particularly in Europe and China^18^, still believe that prophylactic drainage may remove collected fluid, thus reducing the risk of intra-abdominal infection, favouring early detection of postoperative complications such as intra-abdominal bleeding or anastomotic leakage, and minimize their severity, possibly avoiding reoperation^19,20^.

Despite the above-mentioned recommendations, recent large observational studies in Italy, Spain and Europe^21–25^ report an abdominal drain placement rate after colorectal resection ranging from 40 to 70 per cent, reaching 90 per cent in a recent survey among German and Austrian surgeons^26^, whereas these rates are generally reported below 15 per cent in North America^27,28^.

The aim of the present study was to address the existing gap in knowledge by evaluating the impact of the omission of abdominal drains on early adverse events in patients who underwent elective colorectal surgery. Data were used from two prospective open-label observational multicentre studies of the Italian ColoRectal Anastomotic Leakage (iCral) study group^24,25^.

## Methods

### Study design

This was a retrospective propensity score-matched analysis (PSMA) of patients who had undergone colorectal surgery for malignant and benign diseases enrolled in two consecutive studies upon explicit inclusion/exclusion criteria, in 78 surgical centres in Italy from January 2019 to September 2021: iCral2^24^ and iCral3^25^.

### Patient population and data collection

The inclusion criteria were: ASA class I, II or III; elective or delayed urgency setting (defined as >48 h from admission in iCral2 and >24 h from admission in iCral3); patient’s written informed consent for inclusion in the study and processing of sensitive data. The exclusion criteria were pregnancy, hyperthermic chemotherapy (HIPEC) for carcinomatosis and incomplete data. The iCral2 study excluded patients with a protective stoma proximal to the anastomosis; conversely, these patients were included in the iCral3 study. Both studies were conducted in accordance with the Declaration of Helsinki and guidelines for good clinical practice E6 (R2). The study protocols were approved by the ethics committee of the coordinating centre (Marche Regional Ethics Committee (CERM) 2018/334 released on 28 November, 2018 for iCral2 and 2020/192 released on 30 July, 2020 for iCral3) and registered at clinicaltrials.gov (NCT03771456 for iCral2 and NCT04397627 for iCral3). Subsequently, all other centres were authorized to participate by their local ethics committees. Due to the retrospective nature of the current analysis, no specific authorization was requested.

**Fig. 1:**
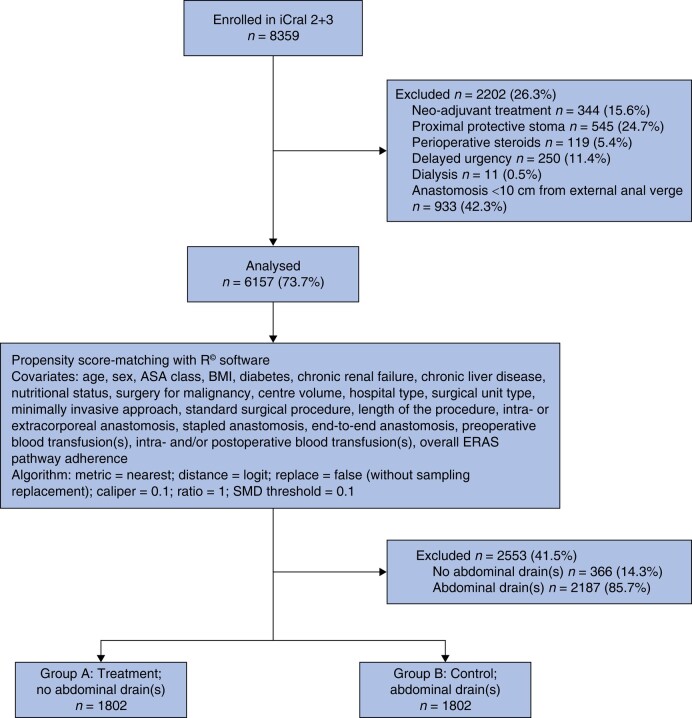
Study flow chart according to the reporting and guidelines in propensity score analysis^29^ iCral, Italian ColoRectal Anastomotic Leakage study group; ERAS, enhanced recovery after surgery; SMD, standardized mean difference.

To control for data imbalance derived from several treatment confounders, the present PSMA study included 6157 patients (73.7 per cent) out of 8359 in the parent studies, based on explicit exclusion criteria: any anastomosis located <10 cm from the anal verge, any anastomosis protected by a proximal stoma, delayed urgency, neo-adjuvant therapy, perioperative steroids and dialysis (*Fig. 1*). The variables and outcomes recorded in the PSMA study population are shown in *Tables 1* and *2*. To optimize the effectiveness of PSMA by reducing the number of unmatched cases, continuous variables were categorized according to their median values.

**Table 1:** Descriptive analysis of the variables considered in the 6157 patients evaluated by the Italian ColoRectal Anastomotic Leakage study group (iCral)

	Overall (*n* = 6157)	No drain(s) (*n* = 2168)	Drain(s) (*n* = 3989)	*P* *
**Age (years)**				
< 70	3133 (50.9)	1112 (51.3)	2021 (50.1)	0.640
≥ 70	3024 (49.1)	1056 (48.7)	1968 (49.3)	
**Sex**				
Male	3205 (52.0)	1059 (48.2)	2146 (53.8)	<0.010
Female	2952 (48.0)	1109 (51.2)	1843 (46.2)	
**ASA class**				
I–II	3916 (63.6)	1455 (67.1)	2461 (61.7)	
III	2241 (36.4)	713 (32.9)	1528 (38.3)	<0.010
**BMI (kg/m^2^)**				
≤ 25.25	3092 (50.2)	1138 (52.5)	1954 (49.0)	
> 25.25	3065 (49.8)	1030 (47.5)	2035 (51.0)	0.010
**Diabetes**				
Yes	917 (14.9)	282 (13.0)	635 (15.9)	
No	5240 (85.1)	1886 (87.0)	3354 (84.1)	<0.010
**Chronic renal failure**				
Yes	256 (4.2)	93 (4.3)	163 (4.1)	
No	5901 (95.8)	2075 (95.7)	3826 (95.9)	0.700
**Chronic liver disease**				
Yes	66 (1.1)	16 (0.7)	50 (1.2)	0.060
No	6091 (98.9)	2152 (99.3)	3939 (98.8)	
**MNA-SF**				
≤ 12	3282 (53.3)	1025 (47.3)	2257 (56.6)	
> 12	2875 (46.7)	1143 (52.7)	1732 (43.4)	<0.010
**Surgery for malignancy**				
Yes	4496 (73.0)	1655 (76.3)	2841 (71.2)	<0.010
No	1661 (27.0)	513 (23.7)	1148 (28.8)	
Diverticular disease	882 (53.1)	290 (13.4)	592 (14.8)	
Endometriosis	45 (2.7)	2 (0.1)	43 (1.1)	
Polyps	318 (19.2)	141 (6.5)	177 (4.4)	
IBD	180 (10.8)	32 (1.5)	148 (3.7)	
Other	236 (14.2)	48 (2.2)	188 (4.7)	
**Mini-invasive surgery**				
No	913 (14.8)	134 (6.2)	779 (19.5)	
Yes	5244 (85.2)	2034 (93.8)	3210 (80.5)	<0.010
Laparoscopic	4441 (84.7)	1815 (83.7)	2626 (65.8)	
Robotic	508 (9.7)	178 (8.2)	330 (8.3)	
Converted	295 (5.6)	41 (1.9)	254 (6.4)	
**Standard procedure**				
Yes	5192 (84.3)	1940 (89.5)	3252 (81.5)	<0.010
Right colectomy	2852 (54.9)	1177 (54.3)	1675 (42.0)	
Left colectomy	2029 (39.1)	684 (31.6)	1345 (33.7)	
Anterior resection	311 (6.0)	79 (3.6)	232 (5.8)	
No	965 (15.7)	228 (10.5)	737 (18.5)	
Transverse colectomy	154 (16.0)	45 (2.1)	109 (2.7)	
Splenic flexure colectomy	218 (22.6)	72 (3.3)	146 (3.7)	
Hartmann reversal	149 (15.4)	24 (1.1)	125 (3.1)	
(Sub) total colectomy	120 (12.4)	24 (1.1)	96 (2.4)	
Other	324 (33.6)	63 (2.9)	261 (6.5)	
**Anastomosis 1**				
Intracorporeal	3964 (64.4)	1779 (82.1)	2185 (54.3)	<0.010
Extracorporeal	2193 (35.6)	389 (17.9)	1804 (45.2)	
**Anastomosis 2**				
Stapled	5460 (88.7)	2043 (94.2)	3417 (85.7)	<0.010
Handsewn	697 (11.3)	125 (5.8)	572 (14.3)	
**Anastomosis 3**				
End-to-end	2467 (40.1)	779 (35.9)	1688 (42.3)	<0.010
Other shape	3690 (59.9)	1389 (64.1)	2301 (57.7)	
**Operation length (min)**				
≤ 170	3169 (51.5)	1265 (58.3)	1904 (47.7)	
> 170	2988 (48.5)	903 (41.7)	2085 (52.3)	<0.010
**Hospital type**				
Met./ac.	4012 (65.2)	1459 (67.3)	2553 (64.0)	0.010
Local/regional	2145 (34.8)	709 (32.7)	1436 (36.0)	
**Unit type**				
Colorectal/oncologic	1107 (18.0)	372 (17.2)	735 (18.4)	
General	5050 (82.0)	1796 (82.8)	3254 (81.6)	0.220
**Centre volume**				
< 4 patients/month	1822 (29.6)	577 (26.6)	1245 (31.2)	
≥ 4 patients/month	4335 (70.4)	1591 (73.4)	2744 (68.8)	<0.010
**Preoperative BT(s)**				
Yes	374 (6.1)	127 (5.9)	247 (6.2)	
No	5783 (93.9)	2041 (94.1)	3742 (93.8)	0.600
**Intra-/postoperative BT(s)**				
Yes	417 (6.8)	114 (5.3)	303 (7.6)	<0.010
No	5740 (93.2)	2054 (94.7)	3686 (92.4)	
**Overall ERAS adherence (%)**				
≤ 75.0	3161 (51.3)	668 (30.8)	2493 (62.5)	
> 75.0	2996 (48.7)	1500 (69.2)	1496 (37.5)	<0.010
Nutritional screening	4170 (67.7)	1628 (75.1)	2542 (63.7)	
Prehabilitation	2386 (38.8)	1097 (50.6)	1289 (32.3)	
Counselling	4073 (66.2)	1716 (79.2)	2357 (59.1)	
Immune enhancing nutrition	1830 (29.7)	854 (39.4)	976 (24.5)	
Antithrombotic prophylaxis	5607 (91.1)	2023 (93.3)	3584 (89.9)	
Antibiotic prophylaxis	5771 (93.7)	2061 (95.1)	3710 (93.0)	
No mechanical bowel preparation	4257 (69.1)	1784 (82.3)	2473 (62.0)	
Preoperative carbohydrates load	3449 (56.0)	1520 (70.1)	1929 (48.4)	
No preanaesthesia	4739 (77.0)	1857 (85.7)	2882 (72.3)	
Standard anaesthesia protocol	4936 (80.2)	1862 (85.9)	3074 (77.1)	
Normothermia	5588 (90.8)	2039 (94.1)	3549 (89.0)	
Goal-directed or restrictive fluid therapy	4738 (77.0)	1816 (83.8)	2922 (73.3)	
Postoperative nausea/vomit prophylaxis	5253 (85.3)	1927 (88.9)	3326 (83.4)	
Multimodal analgesia	5434 (88.3)	2048 (94.5)	3386 (84.9)	
No nasogastric tube	5145 (83.6)	2064 (95.2)	3081 (77.2)	
Minimally invasive surgery	5244 (85.2)	2034 (93.8)	3210 (80.5)	
Urinary catheter < 24–48 h	4746 (77.1)	1971 (90.9)	2775 (69.6)	
Early mobilization	3501 (56.9)	1593 (73.5)	1908 (47.8)	
Early oral feeding	3243 (52.7)	1574 (72.6)	1669 (41.8)	
Predischarge check	4916 (79.8)	2025 (93.4)	2891 (72.5)	

Values are n (%) unless otherwise stated. *Chi square independence test with one degree of freedom; MNA-SF, Mini Nutritional Assessment—Short Form; IBD, inflammatory bowel disease; Intracorporeal, anastomosis performed under visual control through the scope; Extracorporeal, anastomosis performed under direct visual control through an open access; Met./ac., metropolitan/academic; BT, blood transfusion; ERAS, enhanced recovery after surgery items.

### Outcomes

All enrolled patients were followed up for 8 weeks after surgery by local investigators, who were left free to manage the perioperative interval according to their usual local criteria, including any additional exam and time to discharge. Any adverse event was recorded and graded according to Clavien–Dindo^30^ and the Japanese Clinical Oncology Group (JCOG) extended criteria^31^ as well as any reoperation, readmission or death. Anastomotic leakage (AL) was defined according to the international consensus^32^. All the outcomes were calculated at 60 days after surgery.

The primary endpoint was the duration of postoperative hospital stay (LOS, inclusive of any readmission) either dichotomized according to its median value or considered as a continuous variable. The secondary endpoints were: superficial and/or deep surgical site infections (s-d-SSI), defined as drain-specific complications including purulent drainage from superficial incisions, positive culture of fluid or tissue from superficial incisions, pain or tenderness, localized swelling, redness, heat, and/or infections involving deep fascial and muscle layers without dehiscence^33^; deep wound dehiscence; abdominal collection/abscess defined as intraperitoneal postoperative collections that altered the normal postoperative course, requiring either medical, radiological, endoscopic or surgical intervention^33^; SSI defined as s-d-SSI plus abdominal collection/abscess plus deep wound dehiscence; infectious morbidity rate defined as SSI plus pulmonary infections plus urinary infections; AL; overall morbidity rate (any adverse event); major morbidity rate (any adverse event grade > II); reoperation (any unplanned operation) rates; mortality (any death) rates.

### Statistical analysis

This was a retrospective PSMA of two prospective cohorts, with sample sizes calculated and reported in the respective core papers^24,25^. Events per variable guideline were followed^34^. There were no missing data in the database of 6157 patients. The target of estimand was represented by the average treatment effect in the true population of interest (ATT).

A propensity score-matching model^35,36^ was used for the analysis (*Fig. 1*). An adjusted logistic regression was used to estimate the propensity scores of the treatment and control groups. The exposure variable was a treatment that implied no abdominal drain(s) placement in elective colorectal surgery, and 21 covariates, potentially affecting the treatment^37^, were selected: age, sex, ASA class, BMI, diabetes, chronic renal failure, chronic liver disease, nutritional status measured through the Mini Nutritional Assessment—Short Form (MNA-SF)^38^, surgery for malignancy, centre volume, hospital type (academic/metropolitan *versus* local/regional), surgical unit type (general *versus* oncologic/colorectal), mini-invasive surgery, standard surgical procedure, operation length (minutes), intra- or extracorporeal anastomosis, stapled *versus* handsewn anastomosis, end-to-end anastomosis, preoperative blood transfusion(s), intra- and/or postoperative blood transfusion(s), and overall ERAS pathway adherence rates.

To ensure that the treatment groups were balanced^39^, a PSMA using the software ‘R^©^’ (Version 4.2.2, The R Foundation^©^ for Statistical Computing, Vienna, Austria, 2022) was performed. A nearest neighbour approach with a logit distance metric and a caliper of 0.1 to minimize differences between the groups was used as well as adjusted logistic regression to estimate the association between the treatment variable and outcomes.

Balance in the matched groups was assessed by calculating the standardized mean difference (SMD), using a threshold of 0.1 (an SMD less than 0.1 typically indicates a negligible difference between the means of the groups) and the general variance ratio (a variance ratio close to 1 indicates that variances are equal in the two groups). For outcome modelling, an adjusted logistic regression was performed based on a treatment variable represented by no abdominal drain placement in elective colorectal surgery and on the same 21 covariates selected for the PSMA^40^, presenting odds ratios (OR) and 95 per cent c.i. The eventual effect of any unobserved confounder was tested through a sensitivity analysis^41^, using the library ‘SensitivityR5’ of the software R^©^ (Version 4.2.2, The R Foundation^©^ for Statistical Computing, Vienna, Austria, 2022) and presenting the Γ values (each 0.1 increment of Γ values representing a 10 per cent odds of differential assignment to treatment due to any unobserved variable).

## Results

A total of 8359 patients who underwent colorectal resection with anastomosis were enrolled in two consecutive studies upon explicit inclusion/exclusion criteria, in 78 surgical centres in Italy from January 2019 to September 2021: iCral2^24^ and iCral3^25^.

The overall rate of abdominal drain placement after elective colorectal surgery was 64.8 per cent (3989 of 6157 patients). *Tables 1* and *2* provide descriptions of the study covariates and, regarding univariable outcome analysis, drain omission was significantly associated with a lower risk of s-d-SSI, SSI, overall morbidity rate, mortality rate and LOS >6 days. The prevalence characteristics of the 3989 patients in whom abdominal drain(s) were placed are reported in *Table 2*. Drain(s) placement was significantly prevalent in males, ASA III, BMI >25.25 kg/m^2^, diabetes, MNA-SF ≤12, surgery for benign disease open surgery, non-standard procedures (transverse colectomy, splenic flexure colectomy, Hartmann reversal, (sub) total colectomy, other) in comparison to standard procedures (right colectomy, left colectomy, anterior resection), extracorporeal anastomosis, handsewn anastomosis, end-to-end anastomosis, operation length >170 min, local/regional hospitals in comparison to metropolitan/academic hospitals, centre volume < 4 patients/month, intra/postoperative blood transfusion(s), overall ERAS adherence <75 per cent.

**Table 2:** Descriptive analysis of the outcomes considered in the 6157 patients evaluated by the Italian ColoRectal Anastomotic Leakage

	Overall	No drain(s)	Drain(s)	OR (95% c.i.)*
**s-d-SSI**				
Yes	208 (3.4)	52 (2.4)	156 (3.9)	0.60 (0.44–0.83) *P* <0.010
No	5949 (96.6)	2116 (97.6)	3833 (96.1)	Reference
**Deep wound dehiscence**				
Yes	14 (0.2)	6 (0.3)	8 (0.2)	1.38 (0.48–3.99) *P* = 0.550
No	6143 (99.8)	2162 (99.7)	3981 (99.8)	Reference
**Abdominal collection/abscess**				
Yes	87 (1.4)	29 (1.3)	58 (1.6)	0.92 (0.59–1.44) *P* = 0.710
No	6070 (98.6)	2139 (98.7)	3931 (98.5)	Reference
**SSI**				
Yes	290 (4.7)	84 (3.9)	206 (5.2)	0.74 (0.57–0.96) *P* = 0.020
No	5867 (95.3)	2084 (96.1)	3783 (94.8)	Reference
**Infectious morbidity rate**				
Yes	401 (6.5)	125 (5.8)	276 (6.9)	0.82 (0.66–1.02) *P* = 0.080
No	5756 (93.5)	2043 (94.2)	3713 (93.1)	Reference
**Reoperation on**				
Yes	284 (4.6)	101 (4.7)	183 (4.6)	1.02 (0.79–1.30) *P* = 0.900
No	5873 (95.5)	2067 (95.3)	3806 (95.4)	Reference
**LOS**				
≤ 6 days	3966 (64.4)	1683 (77.6)	2283 (57.2)	Reference
> 6 days	2191 (35.6)	485 (22.4)	1706 (42.8)	0.39 (0.34–0.43) *P* <0.010
**Anastomotic leakage**				
Yes	211 (3.4)	67 (3.1)	144 (3.6)	0.85 (0.63–1.14) *P* = 0.280
No	5946 (96.6)	2101 (96.9)	3845 (96.4)	Reference
**Overall morbidity rate**				
Yes	1666 (27.1)	542 (25.0)	1124 (28.2)	0.85 (0.75–0.96) *P* = 0.010
No	4491 (72.9)	1626 (75.0)	2865 (71.8)	Reference
**Major morbidity rate**				
Yes	331 (5.4)	108 (5.0)	223 (5.6)	0.89 (0.70–1.12) *P* = 0.310
No	5826 (94.6)	2060 (95.0)	3766 (94.4)	Reference
**Mortality rate**				
Yes	56 (0.9)	10 (0.5)	46 (1.2)	0.40 (0.20–0.79) *P* = 0.010
No	6101 (99.1)	2158 (99.5)	3943 (98.8)	Reference
LOS (days)	6.89(6.08)† (6 (4–7)‡)	5.67(5.57)† (4 (3–6)‡)	7.55(6.24)† (6 (5–8)‡)	

Values are *n* (%) unless otherwise stated. *Univariate ORs estimation with Wolf valuation of the c.i.; †Mean(s.d.). ‡Median (i.q.r.). s-d-SSI, superficial and/or deep surgical site infections; SSI, s-d-SSI plus deep wound dehiscence plus abdominal collection/abscess; Infectious morbidity rate, s-d-SSI plus deep wound dehiscence plus abdominal collection/abscess plus pulmonary infections plus urinary infections; LOS, length of postoperative hospital stay.

**Fig. 2:**
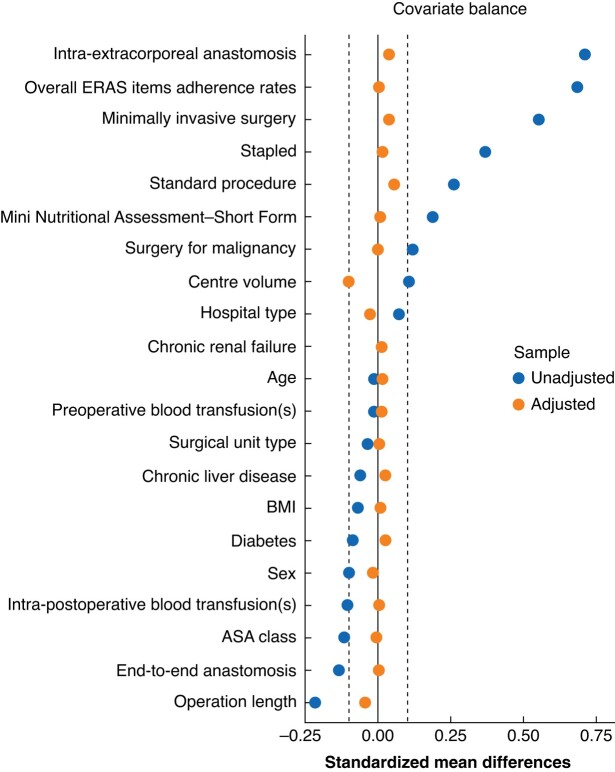
Love plot of covariate standardized mean differences between treatment and control groups before and after matching; the vertical lines represent the interval of ± 0.1 within which balance is considered acceptable ERAS, enhanced recovery after surgery.

For the PSMA, 3604 patients were included, and two groups of 1802 patients were generated (*Fig. 1*): group A (no abdominal drain(s), true population of interest), and group B (abdominal drain(s), control population). This population of 3604 patients included data deriving from 77 (98.7 per cent) of the original 78 centres: group A included data deriving from 60 (77.9 per cent) centres and group B from 75 (97.4 per cent) centres. A good balance between the two groups was achieved, SMD within 0.1 (*Table 3* and *Fig. 2*), with a model variance ratio of 1.0843.

**Table 3:** Variables distribution in treatment and control groups before and after propensity score-matching

	Before propensity score-matching				After propensity score-matching			
Covariates	No drain(s)	Drain(s)	*P* *	SMD	No drain(s)	Drain(s)	*P* *	SMD
	*n* = 2168 (35.2%)	*n* = 3989 (64.8%)			*n* = 1802 (50.0%)	*n* = 1802 (50.0%)		
**Age**								
< 70 years	1112	2021	0.660	−0.01	903	919	0.620	0.02
≥ 70 years	1056	1968	0.660	0.01	899	883	0.620	−0.02
**Sex**								
Male	1059	2146	<0.010	0.01	903	921	0.570	0.02
Female	1109	1843	<0.010	−0.01	899	881	0.570	−0.02
**ASA class**								
I–II	1455	2461	<0.010	−0.11	1192	1188	0.920	−0.005
III	713	1528	<0.010	0.11	610	614	0.920	0.005
**BMI (kg/m^2^)**								
≤ 25.25	1138	1954	<0.010	−0.07	892	898	0.870	0.01
> 25.25	1030	2035	<0.010	0.07	910	904	0.870	−0.01
**Diabetes**								
Yes	282	635	<0.010	0.08	257	241	0.470	−0.03
No	1886	3354	<0.010	−0.08	1545	1561	0.470	0.03
**Chronic renal failure**								
Yes	93	163	0.750	−0.01	78	73	0.740	−0.01
No	2075	3826	0.750	0.01	1724	1729	0.740	0.01
**Chronic liver disease**								
Yes	16	50	0.080	0.05	15	11	0.550	−0.03
No	2152	3939	0.080	−0.05	1787	1791	0.550	0.03
**MNA-SF**								
≤ 12	1025	2257	<0.010	0.19	901	908	0.840	0.01
> 12	1143	1732	<0.010	−0.19	901	894	0.840	−0.01
**Surgery for malignancy**								
Yes	1655	2841	<0.010	−0.12	1344	1344	1.000	0.00
No	513	1148	<0.010	0.12	458	458	1.000	0.00
**Mini-invasive surgery**								
Yes	2034	3210	<0.010	−0.41	1672	1656	0.350	−0.03
No	134	779	<0.010	0.41	130	146	0.350	0.03
**Standard procedures**								
Yes	1940	3252	<0.010	−0.23	1596	1565	0.130	−0.05
No	228	737	<0.010	0.23	206	237	0.130	0.05
**Anastomosis 1**								
Intracorporeal	1779	2185	<0.010	−0.61	1422	1396	0.310	−0.03
Extracorporeal	389	1804	<0.010	0.61	380	406	0.310	0.03
**Anastomosis 2**								
Stapled	2043	3417	<0.010	−0.29	1681	1675	0.740	−0.01
Handsewn	125	572	<0.010	0.29	121	127	0.740	0.01
**Anastomosis 3**								
End-to-end	779	1688	<0.010	0.13	704	700	0.920	−0.004
Other shape	1389	2301	<0.010	−0.13	1098	1102	0.920	0.004
**Operation length**								
≤ 170 min	1265	1904	<0.010	−0.21	1024	986	0.210	−0.04
> 170 min	903	2085	<0.010	0.21	778	816	0.210	0.04
**Hospital type**								
Met./ac.	1459	2553	0.010	−0.07	1169	1193	0.420	0.03
Local/regional	709	1436	0.010	0.07	633	609	0.420	−0.03
**Unit type**								
Col/onc	372	735	0.230	0.03	327	324	0.930	−0.004
General	1796	3254	0.230	−0.03	1475	1478	0.930	0.004
**Centre volume**								
Low	577	1245	<0.010	0.10	513	432	<0.010	−0.10
High	1591	2744	<0.010	−0.10	1289	1370	<0.010	0.10
**Preoperative BT**								
Yes	127	247	0.640	0.01	112	108	0.830	−0.01
No	2041	3742	0.640	−0.01	1690	1694	0.830	0.01
**Intrapostoperative BT**								
Yes	114	303	<0.010	0.10	106	104	0.940	−0.005
No	2054	3686	<0.010	−0.10	1696	1698	0.940	0.005
**Overall ERAS adherence**								
≤ 75.0%	668	2493	<0.010	0.67	656	657	1.000	0.001
> 75.0%	1500	1496	<0.010	−0.67	1146	1145	1.000	−0.001

*Student's test for proportions. SMD, standardized mean difference; MNA-SF, Mini Nutritional Assessment--Short Form; Intracorporeal, anastomosis performed under visual control through the scope; Extracorporeal, anastomosis performed under direct visual control through an open access; Met./ac., metropolitan/academic; Col/onc: colorectal/oncologic; BT, blood transfusion; ERAS, enhanced recovery after surgery items.

Group A *versus* group B showed a significantly lower risk of LOS >6 days (408 (22.6 per cent) *versus* 575 (31.9 per cent) events; OR 0.60; 95 per cent c.i. 0.51–0.70; *P* < 0.001). Sensitivity analysis for LOS calculated a Γ of 1.5 (*P* upper bound = 0.090), meaning that assuming the probabilities of assignment to the two treatment groups to be different because of unknown and/or unmeasured confounding variables, 50 per cent of patients should have been treated by drain(s) placement instead of omission to alter the significant association between drain(s) omission and LOS <6 days. The overall mean(standard deviation (s.d.)) LOS was 5.77(5.77) days in group A *versus* 6.63(5.70) days in group B (*P* < 0.0001; two tailed Student’s *t* test with equal variances), with a mean difference of 0.86 days in favour of group A.

No difference was recorded between the two groups regarding all the other endpoints: s-d-SSI (OR 0.98; 95 per cent c.i. 0.64–1.48; *P* = 0.900); deep wound dehiscence (OR 2.20; 95 per cent c.i. 0.52–9.30; *P* = 0.280); abdominal collection/abscess (OR 1.13; 95 per cent c.i. 0.64–1.99; *P* = 0.670); SSI (OR 1.15; 95 per cent c.i. 0.82–1.62; *P* = 0.420); infectious morbidity rate (OR 1.21; 95 per cent c.i. 0.90–1.62; *P* = 0.190); AL (OR 0.99; 95 per cent c.i. 0.67–1.46; *P* = 0.950); overall morbidity rate (OR 1.06; 95 per cent c.i. 0.90–1.24; *P* = 0.480); major morbidity rate (OR 1.11; 95 per cent c.i. 0.81–1.52; *P* = 0.500); reoperation rate (OR 1.19; 95 per cent c.i. 0.85–1.66; *P* = 0.300); mortality rate (OR 0.67; 95 per cent c.i. 0.27–1.68; *P* = 0.390).

## Discussion

This study presents data on a retrospective PSMA of a prospective multicentre database comparing drain(s) *versus* no drain(s) placement after elective colorectal surgery. This study involved 78 surgical centres, representing a snapshot of real-life clinical practice in Italy. Abdominal drain(s) placement after elective colorectal surgery was performed in 64.8 per cent of 6157 patients, and the univariable analysis of this population demonstrated a statistically significant association between drain(s) placement and a higher risk of s-d-SSI, SSI, overall morbidity rate, mortality rate and prolonged LOS, confirming the observations of previous studies^4,7,8,11–16^. Conversely, our PSMA showed that omission of drain(s) placement after elective colorectal surgery was significantly associated with a lower risk of LOS >6 days, albeit with a small and not clinically significant reduction of 0.86 days mean difference. No statistically significant association was detected for secondary outcomes.

The main aim of the present analysis was to identify any reason supporting the use of drains by Italian (and European) surgeons following elective colorectal resections; there was no single reason to support their use. While LOS is an important outcome for hospital managers and for costs associated with the care of patients with colorectal diseases, it is of relatively little interest to patients and surgeons compared with other endpoints such as AL, major morbidity rate, reoperation rate and quality of life. This study did not demonstrate any difference in the risk of AL, major adverse events and reoperations. This disproves the possible role of abdominal drain(s) on earlier diagnosis and treatment of AL, for which we have highlighted the role of the joint use of clinical scores, C-reactive protein and procalcitonin^42^. The use of abdominal and pelvic drain(s) will continue to exist in a minority (for example, <20 per cent) of selected patients (low rectal anastomoses, immunocompromised and/or frail patients, heavily contaminated or dirty procedures, excessive blood loss and/or intraoperative complications). However, the routine placement is not supported^43^, and a progressive de-implementation strategy should be actively sought at organizational and surgeon levels^44^.

A recent retrospective PSMA of a prospective international cohort^23^ on the same topic used a ‘full matching’ model, which may result in bias as some observations may not have suitable matches.

The main strength of this large sample size study is that it followed rigorous guidelines for applying PSMA^29,45^, being based on the following items: rigorous patient selection from the parent population, performed upon explicit criteria: to limit data imbalance, several potential confounders related to the surgical procedure (delayed urgency, operations without any abdominal incision/trans-anal procedures) or exclusively impacting on a subgroup of patients (anastomosis located <10 cm from the anal verge, neo-adjuvant therapy, proximal protective stoma, administration of perioperative steroids, patients treated by dialysis) were excluded; a reasoned inclusion of 21 conditioning variables (covariates): hospital type, surgical unit type and centre volume to account for the potential imbalance of multicentre, clustered data; adherence to the ERAS pathway items to account for the potential imbalance of medical, anaesthetic and surgical perioperative management; resections for benign and malignant diseases, mini-invasive or open surgery, standard and non-standard procedures^24^, intracorporeal (anastomosis performed under visual control through the scope) or extracorporeal (anastomosis performed under direct visual control through an open access) anastomoses, stapled or handsewn anastomoses, end-to-end or different fashion anastomoses, and operation length, in relation to the imbalance of the surgical treatment; pre- and intrapostoperative blood transfusion(s) to account for transfusion-related morbidity rate^46^; age, sex, ASA class, body mass index, diabetes, chronic renal failure, chronic liver disease, and Mini Nutritional Assessment–Short Form, to account for patient imbalance; evaluation of the treatment effect through an adjusted multiple regression model including the same 21 covariates used for matching^40^; a clear, sheer and restrictive balance algorithm (*Fig. 1*); a sensitivity analysis for unmeasured confounders.

Another strength of this study was the large number of enrolled patients in a well-defined time-lapse in a large number of centres, representing a very wide sample of surgical units performing colorectal resections in Italy. Although the multicentre nature of the considered data may be a definite source of clustering bias, it is undoubtedly representative of real-life data.

However, this study has several limitations, and its results should be interpreted with caution. First, several controversial risk factors were not measured or recorded in the parent studies: single surgeon’s experience^47^, material, type and time to removal of drain(s)^48^, and indication (routine or selective) for drain(s) placement^23^. Second, although a sensitivity analysis of unmeasured confounders has been conducted, potential residual unknown factors and the inability to rule out potential measurement errors by the participating investigators, may have had an impact on the results.

This study confirms that abdominal drain(s) placement after elective colorectal surgery is linked to a slightly prolonged non-clinically relevant LOS, without influencing anastomotic leakage, major morbidity rate and reoperation rate. Abdominal drains should not be routinely used in elective colorectal surgery.

## Collaborators

Assistance with the study: iCral study group co-investigators: Stefano Mancini^26^, Gian Luca Baiocchi^27^, Roberto Santoro^28^, Walter Siquini^29^, Gianluca Guercioni^3^, Massimo Basti^30^, Corrado Pedrazzani^31^, Mauro Totis^32^, Alessandro Carrara^33^, Andrea Lucchi^34^, Maurizio Pavanello^35^, Andrea Muratore^36^, Stefano D’Ugo^37^, Alberto Di Leo^38^, Giusto Pignata^39^, Ugo Elmore^40^, Gabriele Anania^41^, Massimo Carlini^42^, Francesco Corcione^43^, Nereo Vettoretto^44^, Graziano Longo^45^, Mario Sorrentino^46^, Antonio Giuliani^47^, Giovanni Ferrari^48^, Lucio Taglietti^49^, Augusto Verzelli^50^, Mariantonietta Di Cosmo^51^, Davide Cavaliere^52^, Marco Milone^53^, Stefano Rausei^54^, Giovanni Ciaccio^55^, Giovanni Tebala^56^, Giuseppe Brisinda^57^, Stefano Berti^58^, Paolo Millo^59^, Luigi Boni^60^, Mario Guerrieri^61^, Roberto Persiani^62^, Dario Parini^63^, Antonino Spinelli^64^, Michele Genna^65^, Vincenzo Bottino^66^, Andrea Coratti^67^, Dario Scala^68^, Umberto Rivolta^69^, Micaela Piccoli^70^, Carlo Talarico^71^, Franco Roviello^72^, Alessandro Anastasi^73^, Giuseppe Maria Ettorre^74^, Mauro Montuori^75^, Pierpaolo Mariani^76^, Nicolò de Manzini^77^, Annibale Donini^78^, Mariano Fortunato Armellino^79^, Carlo Feo^80^, Silvio Guerriero^81^, Andrea Costanzi^82^, Federico Marchesi^83^, Moreno Cicetti^84^, Paolo Ciano^2^, Michele Benedetti^2^, Leonardo Antonio Montemurro^2^, Maria Sole Mattei^2^, Elena Belloni^2^, Daniela Apa^2^, Matteo Di Carlo^2^, Elisa Bertocchi^7^, Gaia Masini^7^ Amedeo Altamura^8^, Francesco Rubichi^8^, Desirée Cianflocca^10^, Marco Migliore^10^, Diletta Cassini^11,12^, Lorenzo Pandolfini^13^, Alessandro Falsetto^13^, Antonio Sciuto^14^, Ugo Pace^15^, Andrea Fares Bucci^15^, Francesco Monari^16^, Grazia Maria Attinà^17^, Angela Maurizi^18^, Michele Simone^19^, Francesco Giudici^20^, Fabio Cianchi^20^, Bruno Sensi^21^, Alessandra Aprile^22^, Domenico Soriero^22^, Andrea Scarinci^23^, Gabriella Teresa Capolupo^24^, Valerio Sisti^25^, Marcella Lodovica Ricci^25^, Andrea Sagnotta^26^, Sarah Molfino^27^, Pietro Amodio^28^, Alessandro Cardinali^29^, Simone Cicconi^3^, Irene Marziali^3^, Diletta Frazzini^30^, Cristian Conti^31^, Nicolò Tamini^32^, Marco Braga^32^, Michele Motter^33^, Giuseppe Tirone^33^, Giacomo Martorelli^34^, Alban Cacurri^34^, Carlo Di Marco^35^, Patrizia Marsanic^36^, Nicoletta Sveva Pipitone Federico^36^, Marcello Spampinato^37^, Lorenzo Crepaz^38^, Jacopo Andreuccetti^39^, Ilaria Canfora^39^, Giulia Maggi^40^, Matteo Chiozza^41^, Domenico Spoletini^42^, Rosa Marcellinaro^42^, Umberto Bracale^43^, Roberto Peltrini^43^, Maria Michela Di Nuzzo^43^, Emanuele Botteri^44^, Simone Santoni^45^, Massimo Stefanoni^46^, Giovanni Del Vecchio^47^, Carmelo Magistro^48^, Silvia Ruggiero^49^, Arianna Birindelli^49^, Andrea Budassi^50^, Daniele Zigiotto^51^, Leonardo Solaini^52^, Giorgio Ercolani^52^, Giovanni Domenico De Palma^53^, Silvia Tenconi^54^, Paolo Locurto^55^, Antonio Di Cintio^56^, Maria Michela Chiarello^57^, Maria Cariati^57^, Andrea Gennai^58^, Manuela Grivon^59^, Elisa Cassinotti^60^, Monica Ortenzi^61^, Alberto Biondi^62^, Maurizio De Luca^63^, Francesco Carrano^64^, Francesca Fior^65^, Antonio Ferronetti^66^, Giuseppe Giuliani^67^, Graziella Marino^68^, Camillo Leonardo Bertoglio^69^, Francesca Pecchini^70^, Vincenzo Greco^71^, Roberto Piagnerelli^72^, Giuseppe Canonico^73^, Marco Colasanti^74^, Enrico Pinotti^75^, Roberta Carminati^76^, Edoardo Osenda^77^, Luigina Graziosi^78^, Ciro De Martino^79^, Giovanna Ioia^79^, Fioralba Pindozzi^80^, Lorenzo Organetti^81^, Michela Monteleone^82^, Giorgio Dalmonte^83^, Gabriele La Gioia^84^.

From: ^26^General & Oncologic Surgery Unit, San Filippo Neri Hospital, ASL Roma 1; ^27^General Surgery Unit 3, Department of Clinical and Experimental Sciences, University of Brescia; ^28^General Oncologic Surgery Unit, Belcolle Hospital, Viterbo; ^29^General Surgery Unit, S. Lucia Hospital, Macerata; ^30^General Surgery Unit, Spirito Santo Hospital, Pescara; ^31^General & HPB Surgery Unit, University Hospital, Verona; ^32^Colorectal Surgery Unit, San Gerardo Hospital, ASST Monza; ^33^1st General Surgery Unit, S. Chiara Hospital, Trento; ^34^General Surgery Unit, ‘Ceccarini’ Hospital, Riccione (RN); ^35^General Surgery Unit, AULSS2 Marca Trevigiana, Conegliano Veneto (TV); ^36^General Surgery Unit, ‘E. Agnelli’ Hospital, Pinerolo (TO); ^37^General Surgery Unit, ‘V. Fazzi’ Hospital, Lecce; ^38^General and Minimally Invasive Surgery Unit, San Camillo Hospital, Trento; ^39^2nd General Surgery Unit 2, Spedali Civili di Brescia; ^40^Gastroenterologic Surgery Unit, IRCCS S. Raffaele Hospital, Milano; ^41^General & Laparoscopic Surgery Unit, University Hospital, Ferrara; ^42^General Surgery Unit, S. Eugenio Hospital, ASL Roma 2; ^43^General Oncologic and Mininvasive Surgery Unit, ‘Federico II’ University, Napoli; ^44^General Surgery Unit, Spedali Civili of Brescia, Montichiari (BS); ^45^General Surgery Unit, Policlinico Casilino, Roma; ^46^General Surgery Unit, Latisana-Palmanova Hospital, Friuli Centrale University (UD); ^47^General Surgery Unit, S. Carlo Hospital, Potenza; ^48^General Oncologic and Mininvasive Surgery Unit, Great Metropolitan Niguarda Hospital, Milano; ^49^General Surgery Unit, ASST Valcamonica, Esine (BS); ^50^General Surgery Unit, Profili Hospital, Fabriano (AN); ^51^General & Upper GI Surgery Unit, University Hospital, Verona; ^52^General & Oncologic Surgery Unit, AUSL Romagna, Forlì (FC); ^53^General & Endoscopic Surgery Unit, ‘Federico II’ University, Napoli; ^54^General Surgery Unit, Gallarate Hospital (VA); ^55^General Surgery Unit, S. Elia Hospital, Caltanissetta; ^56^General Surgery Unit, S. Maria Hospital, Terni; ^57^General Surgery Unit, San Giovanni di Dio Hospital, Crotone; ^58^General Surgery Unit, ASL 5 Liguria POLL, La Spezia; ^59^General Surgery Unit, ‘U. Parini’ Regional Hospital, Aosta; ^60^General Surgery Unit, Fondazione IRCCS Ca’ Granda, Policlinico Maggiore Hospital, Milano; ^61^Surgical Clinic, Torrette Hospital, University of Ancona; ^62^General Surgery Unit, Fondazione Policlinico Universitario Agostino Gemelli IRCCS, Roma; ^63^General Surgery Unit, S. Maria della Misericordia Hospital, Rovigo; ^64^Colorectal Surgery Unit, Humanitas University, Rozzano (MI); ^65^General & Bariatric Surgery Unit, University Hospital, Verona; ^66^General & Oncologic Surgery Unit, Evangelico Betania Hospital, Napoli; ^67^General Surgery Unit, Misericordia Hospital, Grosseto; ^68^Abdominal Oncologic Surgery Unit, Basilicata Oncologic Hospital, Rionero in Vulture (PZ); ^69^General Surgery Unit, Fornaroli Hospital, ASST Ovest Milanese, Magenta (MI); ^70^General Surgery Unit, Civil Hospital, Baggiovara (MO); ^71^General Surgery Unit, Villa dei Gerani Hospital, Vibo Valentia (VV); ^72^Surgical Clinic, University of Siena; ^73^General Surgery Unit, San Giovanni di Dio Hospital, Firenze; ^74^General & Transplant Surgery Unit, San Camillo-Forlanini Hospital, Roma; ^75^General & Mininvasive Surgery Unit, S. Pietro Hospital, Ponte San Pietro (BG); ^76^General Surgery Unit, Pesenti Fenaroli Hospital, Alzano Lombardo (BG); ^77^Surgical Clinic, University of Trieste; ^78^General & Emergency Surgery Unit, University of Perugia; ^79^General & Emergency Surgery Unit, S. Giovanni di Dio e Ruggi d’Aragona Hospital, Salerno; ^80^General Surgery Unit, Delta Hospital, Lagosanto (FE); ^81^General Surgery Unit, ‘F. Murri’ Hospital, Fermo; ^82^General Surgery Unit, S. Leopoldo Hospital, Merate (LC); ^83^Surgical Clinic, University of Parma; ^84^General Surgery Unit, S. Maria della Misericordia Hospital, Urbino (PU); Italy.

## Data Availability

Data are available upon reasonable request from M.Cat., iCral Study Group coordinator (e-mail: marco.catarci@aslroma2.it).
